# Molecular Detection and Genetic Diversity of *Toxoplasma gondii* Oocysts in Cat Faeces from Klang Valley, Malaysia, Using B1 and REP Genes in 2018

**DOI:** 10.3390/pathogens9070576

**Published:** 2020-07-16

**Authors:** Mohammed Nasiru Wana, Mohamad Aris Mohd Moklas, Malaika Watanabe, Ngah Zasmy Unyah, Sharif Alhassan Abdullahi, Ashraf Ahmad Issa Alapid, Norshariza Nordin, Rusliza Basir, Roslaini Abd Majid

**Affiliations:** 1 Department of Medical Microbiology and Parasitology, Faculty of Medicine and Health Sciences, Universiti Putra Malaysia, Serdang, Selangor 43400, Malaysia; mwnasiru@atbu.edu.ng (M.N.W.); ngah@upm.edu.my (N.Z.U.); sharifosis@gmail.com (S.A.A.); asalapid82@gmail.com (A.A.I.A.); 2 Department of Biological Sciences, Faculty of Science, Abubakar Tafawa Balewa University, Bauchi 740272, Nigeria; 3 Department of Human Anatomy, Faculty of Medicine and Health Sciences, Universiti Putra Malaysia, Serdang, Selangor 43400, Malaysia; rusliza@upm.edu.my; 4 Department of Companion Animal Medicine & Surgery, Faculty of Veterinary Medicine, Universiti Putra Malaysia, Serdang, Selangor 43400, Malaysia; malaika@upm.edu.my; 5 Department of Medical Microbiology and Parasitology, Faculty of Clinical Sciences, Bayero University, Kano 700241, Nigeria; 6 Department of Zoology, Faculty of Science-Alasaba, University of Gharyan, Gharyan 010101, Libya; 7 Department of Biomedical Sciences, Faculty of Medicine and Health Sciences, Universiti Putra Malaysia, Serdang, Selangor 43400, Malaysia; shariza@upm.edu.my; 8 Faculty of Medicine and Defence Health, National Defence University of Malaysia, Kem Sungai Besi, Kuala Lumpur 57000, Malaysia

**Keywords:** oocysts, *Toxoplasma gondii*, B1 gene, REP gene, multicopy-target, cat faeces, genetic diversity, Malaysia, PCR

## Abstract

The major route for *Toxoplasma gondii* (*T. gondii*) infection is through the ingestion of foods contaminated with oocyst from cat faeces. The microscopic detection of *T. gondii* oocysts in cat faeces is challenging, which contributes to the failure of detecting or differentiating it from other related coccidian parasites. This study aims to detect *T. gondii* oocysts in cat faeces using two multicopy-target PCR assays and to evaluate their genetic diversity. Cat faecal (200) samples were collected from pet cats (PCs; 100) and free-roaming cats (FRCs; 100) within Klang Valley, Malaysia, and screened for coccidian oocysts by microscopy using Sheather’s sucrose floatation. PCR assays were performed on each faecal sample, targeting a B1 gene and a repetitive element (REP) gene to confirm *T. gondii* oocysts. Additionally, the PCR amplicons from the REP gene were sequenced to further confirm *T. gondii*-positive samples for phylogenetic analysis. Microscopy detected 7/200 (3.5%) *T. gondii*-like oocysts, while both the B1 gene and the REP gene detected 17/200 (8.5%) samples positive for *T. gondii*. All samples that were microscopically positive for *T. gondii*-like oocysts were also shown to be positive by both B1 and REP genes. The BLAST results sequenced for 16/200 (8.0%) PCR-positive *T. gondii* samples revealed homology and genetic heterogeneity with *T. gondii* strains in the GenBank, except for only one positive sample that did not show a result. There was almost perfect agreement (k = 0.145) between the two PCR assays targeting the B1 gene and the REP gene. This is the first report on microscopic, molecular detection and genetic diversity of *T. gondii* from cat faecal samples in Malaysia. In addition, the sensitivities of either the B1 gene or REP gene multicopy-target PCR assays are suitable for the accurate detection of *T. gondii* from cat faeces.

## 1. Introduction

*Toxoplasma gondii* (*T. gondii*) is a protozoan parasite capable of infecting a wide variety of warm-blooded animals, including humans [1]. The warm-blooded animals serve as the intermediate hosts of the parasite, while felines act as the definitive hosts [2]. Available data indicated that about one-third of the human population in the world is infected with *T. gondii* [1,3]. Seroprevalence rates of *T. gondii* in Malaysia are 44.2% in immunocompetent patients and 57.4% among migrant workers [4,5]. In a recent study, the seroprevalence of *T. gondii* antibodies among pregnant women in Malaysia was found at 34.0% [6]. Wild and domestic cats are the only hosts that can disseminate resistant oocysts from their faeces into the environment [7]. Humans and other animals become infected mainly by ingesting food, water, or vegetables contaminated with *T. gondii* oocysts [8]. Infection, to a lesser extent, can also result from the consumption of undercooked meat containing bradyzoite tissue cysts from animals that were previously infected with *T. gondii* [9,10]. Other less common routes involve transplacental transmission from pregnant women to their foetuses during primary infection [10].

The coccidian parasites have a heteroxenous life cycle that produces cysts, which include seven genera: Toxoplasma, Besnotia, Neospora, Hammondia, Sarcocystis, Isospora and Frenkelia [11]. The oocysts can survive harsh environmental conditions, disinfectants such as sulfuric acid (2%) and sodium hypochlorite solutions [8]. Africa and Asia remain the only continents that are largely unexplored on *T. gondii* oocysts occurrence and its genetic diversity in cat faeces [12]. Among Asian countries and, particularly, Southeast Asia, information of *T. gondii* oocysts shedding rate by domestic cat definitive hosts is unavailable [12]. In Malaysia, several studies were conducted on the *T. gondii* seroprevalence in humans and animals [5,13,14]. However, there is a lack of information on *T. gondii* oocysts occurrence in domestic cat faeces from Malaysia.

Studies conducted worldwide have detected the presence of *T. gondii*-like oocysts in cat faeces by microscopy, mouse bioassay and PCR [15,16,17,18,19,20,21]. Copro-microscopy is a traditional technique that reveals the morphology of *T. gondii* oocysts in cat faeces [8,17,21,22], but its main drawback is that it requires an expert microscopist, is time-consuming and has intrinsic failure to differentiate between similarly related coccidian oocysts [17,21,23]. A bioassay in mice is more sensitive compared to copro-microscopy as it provides information on the presence of the oocysts and its viability [23,24], but it is too expensive, requires live mice, and is time-consuming and difficult to operate on a larger scale during epidemiological studies [25,26]. So far, molecular techniques readily used to detect *T. gondii* oocysts in cat faeces utilize the conventional polymerase chain reaction (PCR), loop-mediated isothermal amplification (LAMP) and quantification polymerase reaction (qPCR), with each technique having its unique advantage [10,23,26]. Copro-PCR assay using a multicopy target gene is more sensitive, specific, time-efficient and robust compared to the other two techniques [27,28]. Hence, this study aims to detect and determine the genetic heterogeneity of the occurrence of *T. gondii* oocysts in pet cat (PC) and free-roaming cat (FRC) faeces from Klang Valley, Malaysia, where the concentration of cats are believed to be higher due to the high number of restaurants and the habit of keeping domestic cats as pets by the populace. Molecular detection of *T. gondii* was carried out using two multicopy-target PCR assays for the B1 gene and the repetitive element (REP) gene, followed by sequencing to confirm and determine the genetic diversity of the *T. gondii*-positive samples.

## 2. Results

### 2.1. Copro-Microscopy

**Figure 1:**
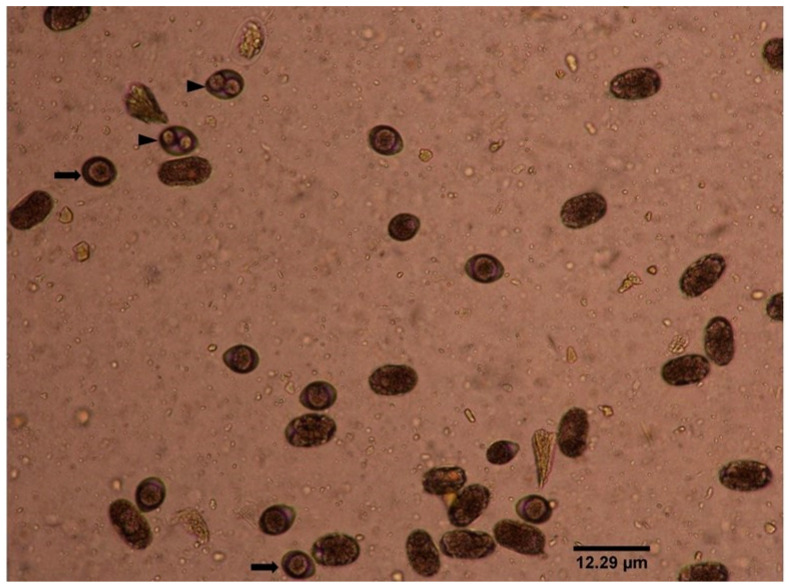
Representative image of *T. gondii*-like oocysts in Sheather’s sucrose solution in infected cat faeces. The symbol with the arrow-head is sporoluted, whereas an arrow is an unsporoluted oocyst. (Magnification 400×).

Using copro-microscopy, 200 cat faeces were screened for the presence of *T. gondii*-like oocysts, out of which 7/200 (3.5%) were detected as positive. More *T. gondii*-like oocysts were found in FRCs (5/100, 5.0%) compared to PCs (2/100, 2.0%). A representative image of a *T. gondii*-like oocyst is presented in Figure 1. The percentage of the total number of *T. gondii*-like oocysts detected by copro-microscopy in both PCs and FRCs is also shown in Table 1.

### 2.2. Copro-PCR Assays

**Figure 2:**
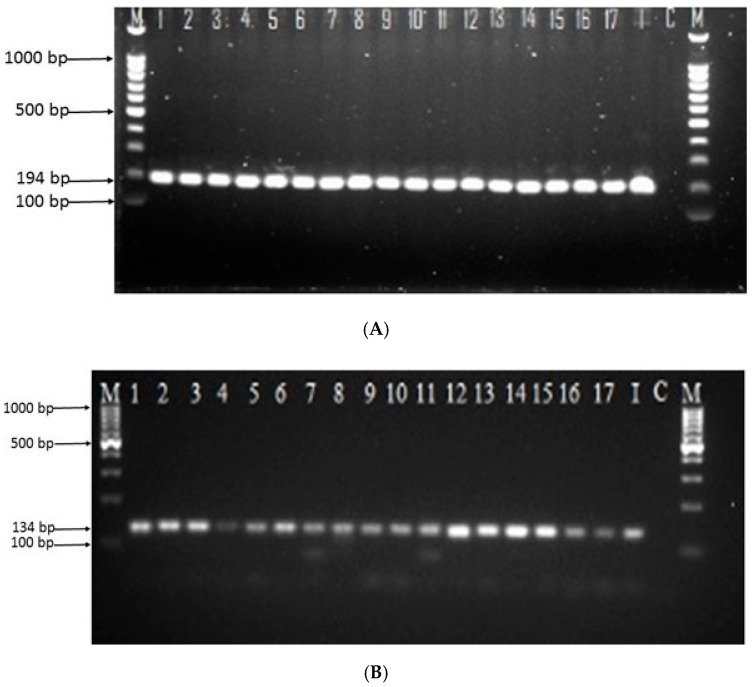
Detection of *T. gondii* oocysts nucleic acid amplified by (**A**) the B1 gene and (**B**) the REP gene. The product was resolved in 1.5% agarose stained with Redsafe. Lane M: 100 bp DNA molecular ladder (Thermo Scientific, GeneRuler, Washington, DC, USA); Lanes 1–17: Malaysian isolates; Lane I reference strains of type I (RH); Lane C: negative control (all PCR reaction mixtures without DNA template).

For the two multicopy-target copro-PCR assays, oocyst DNA was extracted from the 200 faecal samples that were either positive or negative with the copro-microscopy. The extracted DNA was found to have good purity, but with varying concentration, ranging between 90 to 165 ng/µL. The primers used for the amplification of copro-PCR were the B1 gene and the REP gene. A total of 17/200 (8.5%) of the *T. gondii* oocysts were successfully amplified with both primer sequences (Figure 2A,B). The 7/200 (3.5%) that were positive with copro-microscopy were all amplified using both B1 gene and REP gene multicopy-target copro-PCR assays, including additional 10 faecal samples that were *T. gondii*-like oocyst-negative. Thirteen out of the 17 copro-PCR positive faecal samples came from FRCs (13/100, 13.0%), which showed higher *T. gondii* oocysts detected compared with PCs (4/100, 4.0%). Although copro-microscopy and copro-PCR assays differ in the number of *T. gondii* oocysts detected in cat faeces, the two techniques detected lower *T. gondii* oocysts in PC faeces compared with FRC faeces. There was no statistically significant relationship between the two groups of PCs and FRCs (*p* > 0.99) infected with *T. gondii*. Further, an odds ratio of 1.3 indicates that there were higher chances to detect *T. gondii* with the two multicopy-target copro-PCR assays compared to copro-microscopy. The percentage of the two PCR assay detection in both PCs and FRCs is illustrated in Table 1. An almost perfect agreement (k = 0.145; 95% confidence interval, −0.285 to 0.285: *p* < 0.05) was observed between the two multicopy-target PCR assays.

### 2.3. DNA Sequence Results

**Figure 3:**
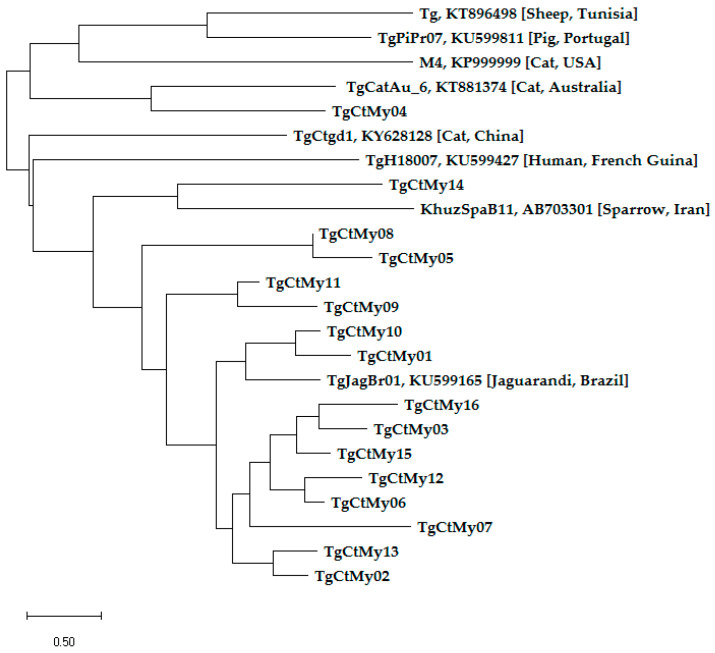
Phylogenetic tree of *T. gondii* isolates in cat samples from Malaysia (TgCtMy01-TgCtMy16) depicting the evolutionary relationship between taxa derived from the REP gene and 8 *T. gondii* reference strains. These *T. gondii* reference strains (Tg, TgCtd1, KhuzSpaB11, TgCatAu_6, M4, TgTagBr01, TgH18007, TgPiPr07) were from Tunisia, China, Iran, Australia, the USA, Brazil, French Guinea and Portugal as representative(s) from each continent (Africa, Asia, Australia, North America, South America and Europe), based on the available information in the Genbank. The evolutionary history for the REP gene was inferred using the neighbour-joining method [29]. The optimal tree with the sum of branch length = 26.95290385 is shown. The tree is drawn to scale, with branch lengths in the same units as those of the evolutionary distances used to infer the phylogenetic tree. The evolutionary distances were computed using the maximum likelihood method [30] and are in the units of the base substitutions per site. This analysis involved 24 nucleotide sequences. Codon positions included were 1st + 2nd + 3rd noncoding. All ambiguous positions were removed for each sequence pair (pairwise deletion option). There was a total of 677 positions in the final dataset. Evolutionary analysis was conducted in MEGA X [31]. The *T. gondii* sequences in this study were designated from TgCtMy01 to TgCtMy16, and their interpretations are presented in Table 2. The 8 *T. gondii* reference strains deposited in Genbank, as shown in Figure 3, are indicated by their isolate name and accession number, while the host and country of origin are given in brackets.

The BLAST results sequenced for the 16/200 (8.0%) PCR-positive *T. gondii* samples were identical with *T. gondii* strains, except for one sample in which the sequenced DNA product (TTTGGGCGGCTGGCACGAGAGAGTCGGAGAGGGAGAAGAAGGTTCCGGGTTGGCTGGTTTTCCTGGAGGGGGGGAAAGAGACACCGGAATGCGATCCCCACAAGACGAAAA) submitted to GenBank did not yield a result (Table 2). This may likely have resulted from a low amount of DNA or low quality of sequence for analysis. Further, the forward and reverse primer sequences were concatenated and verified using BioEdit Software (https://bioedit.software.informer.com/7.2/). The whole gene sequence for each sample was BLAST in NCBI, and the FASTA sequence data were aligned with Clustal W. The homology sequences that were 90%–100% identical to REP (A146527) when BLAST in NCBI was selected for the alignment. The results revealed a wide genetic diversity of *T. gondii* in Malaysia, with some samples closely related to other identified *T. gondii* sequences deposited in the Genbank from different regions of the world. The simple phylogenetic tree was constructed using MEGA X software, as shown in Figure 3.

## 3. Discussion

To the author’s knowledge, this is the first report of *T. gondii* oocysts detected directly from cat faeces in Malaysia using both copro-microscopy and copro-PCR techniques. The previous studies conducted in Malaysia on *T. gondii* in the last decade (2008 to 2018) were mainly serological tests for detection of the presence of antibodies in the blood of humans or animals [4,5,32,33,34,35,36,37,38,39,40,41,42,43]. Others searched for new innovative diagnostic approaches and vaccine development for *T. gondii* [44,45,46,47,48], and characterization of *T. gondii* genotype studies on free-range ducks and wild boars [49,50].

The age of the cat determines the rate of *T. gondii* oocysts shedding into the environment. As previously reported [9], young cats shed more oocysts (1–3 weeks) compared to adults. In the present study, two-thirds of the population of PCs at the Universiti Veterinary Hospital (UVH), Universiti Putra Malaysia (UPM), were adults based on weight and size [51] whereas, the ages of the FRCs were unknown. It is likely that the *T. gondii* oocysts detected in FRC faeces comes from young cats [52].

Microcopy is a simple tool, easy to use and inexpensive [23]. The copro-microscopic technique identifies the morphology of the parasite in cat faeces as sporulated or unsporulated [17]. However, the technique has many limitations, which among others, in this study, include failure to detect the *T. gondii* oocysts if they are low in number. Sheather’s sucrose solution, either prepared locally or purchased commercially, has higher specific gravity (1.27) compared with saturated salt solutions such as sodium nitrate (specific gravity 1.18). In the present study, the rate of oocyst recovery by sucrose flotation is similar to the previous findings [28,53,54]. In contrast, only limited studies have reported *T. gondii* oocyst recovery with sodium nitrate solution [55]. Further, this advantage of higher specific gravity by Sheather’s sucrose [28] makes it a reliable and efficient flotation solution to recover and purify *T. gondii* oocysts that can float with minimal distortion [8]. The previous findings were inconsistent due to the variation in the microscopic technique used for the *T. gondii* oocyst detection [8,26]. Nevertheless, in the present study, copro-microscopy detected *T. gondii*-like oocysts in cat faeces of 3.5% (7/200), which is higher than 0.39%, 0.58% and 2.56% reported from Switzerland, Germany and Italy, respectively, [16,19,28]. In addition, previous copro-microscopic detection of *T. gondii*-like oocysts in cat faeces from the USA (California), Canada, Finland and Egypt had documented 0.9%, 1.3%, 1.5% and 0.63%, respectively [18,22,56,57], which is lower when compared to the present study. Also, findings from 16 European countries, which is so far the largest (24,106 cat faeces) study of copro-microscopic detection of *T. gondii*-like oocysts, recorded 0.11% [21]. This result is still low compared with this study. In contrast, the present finding of *T. gondii*-like oocysts in cat faeces from Malaysia of 3.5% with copro-microscopy is lower than 6.0% in the USA (Virginia), 19.0% in Ethiopia and 19.3% in Thailand [17,27,58], while in other studies, *T. gondii*-like oocysts were not detected in cat faeces through copro-microscopy in countries such as Spain, Canada, China, Italy, China (Beijing), Iran, Turkey and Columbia [20,24,25,59,60,61,62,63]. However, such variation in copro-microscopic detection of *T. gondii*-like oocysts was shaped by many factors such as the density of the cat population [19,53,64], the period of shedding oocysts [20,60,61,65], seroprevalence status of the cat [56,58,62], and geographical location [21]. In this study, the prevalence rate of 3.5% *T. gondii*-like oocysts detected in cats faeces may likely be due to the high density of FRCs, with abundant rodents that keep a perpetual life cycle. Thus, a lower number of *T. gondii*-like oocyst-positive samples detected by copro-microscopy in this study allows more cases to go undetected and may likely increase a greater spread of infection in humans and animals [15].

The copro-PCR is a robust technique, sensitive and accurate, which can detect *T. gondii* oocysts nucleic acid in cat faeces [17,28], although the presence of contaminants such as humic acid, clays and polysaccharides in the soil may sometimes prevent proper amplification [66]. This study used multicopy-target B1 gene and REP gene sequences, which are well established, and tested for the detection of *T. gondii* nucleic acid in various laboratories [22,64,66,67,68,69]. The present study revealed that both PCR/B1 gene and PCR/REP gene performed equally at the same rate by amplifying 17/200 (8.50%) samples (Figure 2A,B). In addition, the statistical comparison between the two multicopy-target PCR assays in this study indicated almost perfect agreement. The performance of these two multicopy-target PCR assays was relatively higher when compared to previous studies of *T. gondii* oocyst nucleic acid on cat faeces performed with the same set of primers [17,18,21,27,69]. However, differences in DNA extraction methods or use of the correct amount of BSA in downstream applications could have caused the differences with previous studies.

The majority of the faecal samples that positive for *T. gondii* with copro-PCR showed negative with copro-microscopy. This may be due to the fact that copro-PCR could possibly detect low numbers (1–5) of *T. gondii* oocysts in environmental samples [8], while the copro-microscopy detection threshold is 1000 oocysts/g of faeces [70]. Therefore, faecal samples that were *T. gondii* positive using copro-PCR but negative by copro-microscopy may likely have an insufficient number of oocysts [71].

The present study found the two multicopy-target copro-PCR assays more sensitive compared to copro-microscopy in the detection of *T. gondii* oocysts. This is in accordance with other studies, which also reported high sensitivities of the copro-PCR assay [17,20,21,26]. Molecular techniques have been proven to be species-specific, have fewer chances of false-positives and can detect fewer *T. gondii* oocysts in any environmental matrices [8,26]. Further, the current study found FRCs shedding more *T. gondii* oocysts was statistically higher than PCs. This finding is in agreement with previous studies that found copro-PCR to be more sensitive and species-specific [8,26,64]. FRCs are constantly infected as they consumed rodents [53] and other food materials contaminated with *T. gondii,* which placed them as vulnerable at all times to shed more oocysts as the effect of infection and reinfection continues indefinitely [54,72,73,74,75]. *T. gondii* oocysts may be the main source of human and animal infection in Klang Valley, with defecation sites being the hot spots for infection [64,73,74]. Felines are the only definitive host of *T. gondii*, and the detection of oocysts in their faeces is an indication of a source of human and animal infection [8]. In contrast to other previous studies on molecular detection of *T. gondii* oocyst DNA in cat faeces [11,21], this present study did not detect other coccidian parasites when species-specific multicopy-target PCR assays were used. However, this study area is a small setting; however, high numbers of *T. gondii* oocysts occurrence were detected. Other areas should be surveyed to determine the spread of *T. gondii* oocysts at the national level.

The sequenced BLAST results from PCR-positive samples in this study confirmed all the amplified DNA were of the *T. gondii* strain, except for a single positive DNA sample that failed to show a result (Table 2). This result was achieved due to the high copy of the REP gene that was between 200–300 copies, which is highly species-specific, sensitive and conserved in the genome of *T. gondii* [28,55,67,68]. The simple phylogenetic analysis of 16 PCR DNA positive samples sequenced at the REP gene and 8 *T. gondii* reference strains deposited at Genbank from Tunisia, China, Iran, Australia, the USA, Brazil, French Guinea and Portugal revealed a wide genetic diversity of *T. gondii* strains from Malaysia, in which some had shown close similar identities to their siblings in other regions of the world [76,77]. Nevertheless, this needs more investigation. Further, the genetic diversity of *T. gondii* strains was strengthened by the wide distribution of PCs and FRCs in the study area. The present result of a wide genetic diversity of *T. gondii* strains is in agreement with previous findings, which reported genetic diversity of this parasite on different continents [78,79,80].

An important limitation in this study is that it detected the presence of *T. gondii* oocysts in cat faeces only using PCR, and quantification and estimation of *T. gondii* oocysts in cat faeces are influenced by qPCR [55]. The genetic heterogeneity of *T. gondii* oocysts, such as the B1 gene, in cat faeces from Malaysia, remains to be determined. Anther limitation observed in this study was the small sample size. To provide accurate data of *T. gondii* occurrence in Malaysia, wild and domestic cat faecal samples from different locations should be included. Since only randomly sampled PC and FRC faeces were used in the present study, feral and sheltered cat faeces are also important because they are also sources of *T. gondii* infection. Hence, the occurrence of *T. gondii* in this group of cats needs to be defined to curtail the spread of infection.

## 4. Materials and Methods

### 4.1. Study Area

**Figure 4:**
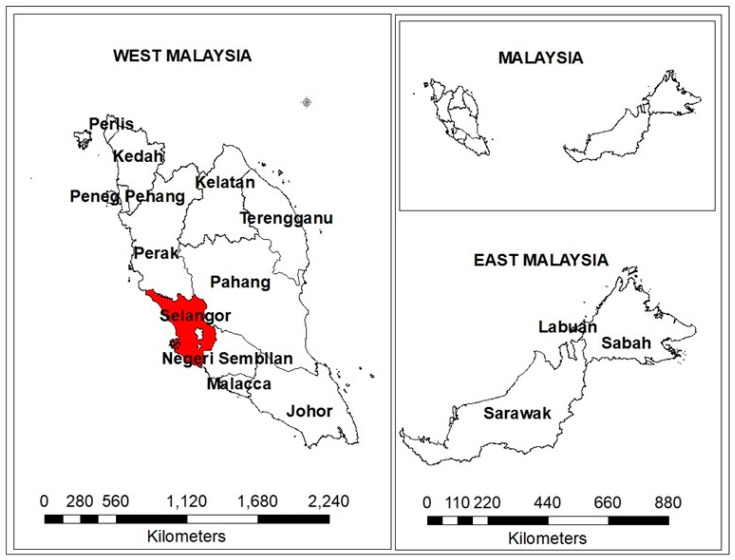
Map of Malaysia showing the study area Klang Valley located within Selangor state coloured red.

The present study was conducted on cat faeces collected in Klang Valley, Malaysia, which is a cosmopolitan city located within Selangor state, a part of West Malaysia (Figure 4). Klang Valley is a densely populated area, with some pockets of empty land covered with bushes close to human settlements. It has an altitude of 249 m (3°4′25.8168″ N 101°31′6.0564″ E). The climate is a tropical rainforest with warm and dry summers throughout the year. The average annual rainfall is 2369 mm, with an average relative humidity of around 60.2%. (https://www.weather-my.com/en/malaysia/serdang).

### 4.2. Cat Faecal Sample Collection

**Figure 5:**
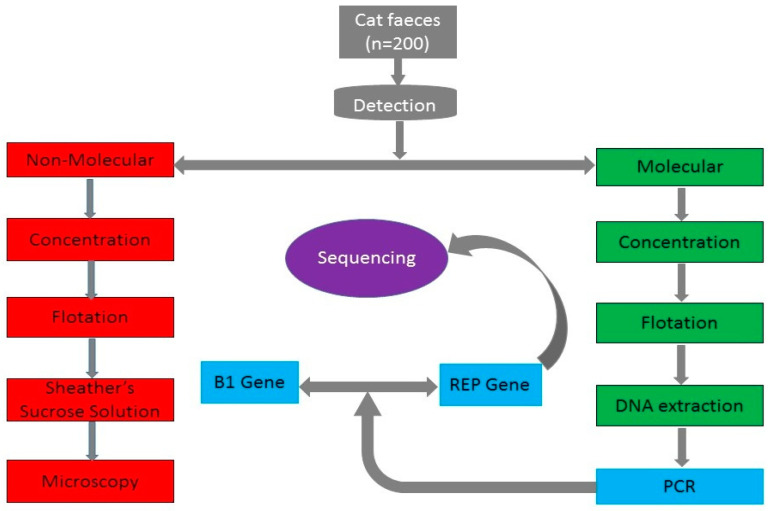
A flow chart describing techniques used to detect *T. gondii* oocyst in cat faeces. Red colour represents the process used for the copro-microscopic identification of *T. gondii* oocysts in cat faeces. Green colour shows the process of DNA extraction from cat faeces, while blue colour indicates the conventional PCR assays using B1 gene and REP gene amplification, which are specific to *T. gondii*. Purple colour represents the sequenced REP gene amplification product.

A convenience sampling method was adopted, where a total of 200 cat faeces samples were collected randomly and analyzed from July 2017 to May 2018 within Klang Valley. One hundred PC faeces were collected from cats admitted at the UVH, UPM, while another set of 100 cat faeces from FRCs in different locations within Klang Valley, Selangor, Malaysia, were also collected. For the purpose of this study, FRCs include cats that were mostly found close to restaurants, local markets and crowded places, but they lack homes and roam freely outside as stray cats. The FRCs also ate all sorts of food, sleep, bred and mingle with people in those areas. Meanwhile, PCs refer to individually owned cats that were fed with commercial feed and strictly kept at homes. The age, sex and dietary intake of all cats were not recorded to avoid bias since most FRCs were rarely found defaecating at the time of collection. Faeces from FRCs were collected fresh at each location once, early in the morning, from initially identified spots as their defecation sites. The FRC faeces were either buried in the soil, in open spaces or on top of grasses. With the help of the attending veterinarian, fresh PC faeces were collected once from newly admitted cats. Each FRC and PC faeces was collected with separate spatula and transferred into a small plastic bag, which was then placed into a screw cap container, labelled and transported to the laboratory for analysis. The outline of this study is shown in Figure 5.

### 4.3. Copro-Microscopic Detection of T. gondii-Like Oocysts in Faeces

Approximately 2–5 g of faeces from each cat faecal sample collected was mixed with 10 mL PBS in a beaker thoroughly. The mixture was passed through 2 layers of gauze. The filtrate was poured into a 15-mL tube and centrifuged at 2000× *g* for 5 min. The supernatant was discarded, and 10 mL of Sheather’s sucrose (Sigma-Aldrich, California, USA) solution (1278 g of sugar, 1000 mL of water and 1.5% formaldehyde; specific gravity of 1.27) was added to the concentrate and centrifuged again at 2000× *g* for 5 min. Further, the sucrose solution was gradually added until it reached brim [26]. The tube was covered with a coverslip and we waited for 10 min for the oocysts to attach to its surface [25,58]. The coverslip was removed and placed into a clean glass slide for microscopic examination. Each faecal sample was prepared in triplicate to eliminate the chances of missed detection. The characteristic features of positive *T. gondii*-like oocysts checked include shape, which is oval or ellipsoidal, two internal distinct sporocysts and a diameter of between 9–15 μm [17,21].

### 4.4. Extraction of Oocyst DNA in Cat’s Faeces

Cat’s faecal sample supernatant (5 mL) prepared in Sheather’s sucrose was collected from the top into a clean 15-mL tube. The tube containing the sample was washed 3 times with double distilled water to concentrate the oocysts at the bottom of the tube. The supernatant was removed, and 400 µL of the sediment was collected and transferred immediately into a clean 2-mL tube and suspended in 400 µL of ASL buffer (QIAmp DNA Stool Mini Kit, Qiagen and Hilden, Germany). This was followed by 3 cycles of freeze–thaw at −80 °C for 5 min to freeze and thaw at 60 °C for 5 min. Subsequently, glass beads (1 mm, Sigma Aldrich, California, USA) were added for oocysts disruption as earlier described [71]. After oocyst disruption, the samples were centrifuged at 11,000× *g* for 3 min. The supernatant was then collected into a clean - mL tube to which 1 mL of buffer ASL was added. The mixture was vortex for 1 min or until the sample was thoroughly homogenised. This was to facilitate an increase in DNA concentration. Thereafter, oocyst DNA extraction was conducted following the manufacturer’s instructions (QIAmp DNA Stool Mini Kit, Qiagen and Hilden, Germany). Elution with AE buffer was performed twice using 100 µL of the first elute. The eluted DNA was measured in a NanoDrop 2000 spectrophotometer (Thermo Scientific, Washington DC, USA). All extracted DNA was stored at −20 °C until later use.

### 4.5. Reference Samples

Reference samples for DNA type I (RH), type II (PRU) and type III (VEG) were generously provided by Prof. Marie-Laure Darde, Institute of Parasitology, and the University of Limoges, France.

### 4.6. Copro-PCR Assays for the Detection of T. gondii in Cat Faeces

The two copro-PCR assays were performed separately with a final volume of 25 µL as reaction mixture, which each contained a multicopy-target sequence of the B1 gene or the REP gene. These primer sequences were previously designed by Burg [67] and Homan et al. [68], as listed in Table 3. The copro-PCR reaction mixtures consist of 12.5 µL of Superhot Master Mix (BIORON GmbH, Germany), 0.2 µM (final concentration) each of forward and reverse primer, 0.1 µg/µL of bovine serum albumin (BSA), 4 µL of DNA template and PCR-grade water (BIORON, GmbH, Hilden, Germany). Amplification was performed in a Bio-Rad Mycycler (Thermal Cycler PCR, California, USA), with an initial denaturation at 95 °C for 3 min. This was followed by 35 cycles of denaturation at 94 °C for 30 s, annealing for each of the 2 primers sequences, as shown in Table 3, for 45 s and extension at 72 °C for 1 min. The final extension was carried out at 72 °C for 10 min. For each reaction mixture, the reference strain of type I (RH) was included as a positive control, while negative control was a complete reaction mixture without a DNA template. The PCR product and 100-bp ladder (BIORON, GmbH, Hilden, Germany) were resolved in 1.5% agarose gel stained with ethidium bromide and visualized with Bio-Rad gel doc XR (Molecular Imager, California, USA).

### 4.7. Sequencing of the REP Gene Amplified PCR Product

The PCR product from the REP gene repetitive element was purified with a QIAquick PCR purification kit, according to the manufacturer’s instructions (Qiagen, GmbH, Hilden, Germany). The eluted DNA collected in a 1.5-mL tube was stored at −20 °C until later use. The purified DNA template and REP gene (forward and reverse primer) were sequenced at MYTACG Bioscience Enterprise, Malaysia.

### 4.8. Statistical Analysis

Statistical analysis was performed using GraphPad Prism (version 7.0) software (GraphPad Software, San Diego, CA, USA). The detection of oocysts occurrence was compared between PCs and FRCs, which was determined by Fischer’s test at 95% confidence levels. Cohen’s kappa (k) was used to determine the agreement between the two multicopy-target PCR assays of B1 and REP gene detection at 95% confidence intervals. Results were interpreted in six scales as follows: kappa < 0: no agreement, kappa between 0.00 and 0.20: slight agreement, kappa between 0.21 and 0.40: fair agreement, kappa between 0.41 and 0.60: moderate agreement, kappa between 0.61 and 0.80: substantial agreement and kappa between 0.81 and 1.00: almost perfect agreement [81].

## 5. Conclusions

In conclusion, the results of this study indicate the presence and wide genetic diversity of *T. gondii* oocysts in cat faeces within Klang Valley, Malaysia. This is the first report of the detection of *T. gondii* oocysts in cat faeces by microscopy, molecular technique and phylogenetic analysis from Malaysia. Further, the results also showed that infected FRCs shed more *T. gondii* oocysts compared to PCs, which may likely be a source of human and animal *T. gondii* infection. Given the lack of information on the estimate of *T. gondii* oocysts in cat faeces from Malaysia to show evidence of large scale soil contamination, further research into the role of *T. gondii* oocyst quantification shedding rates as a source of human and animal infection is needed. Although the role of B1 gene sequence analysis in genetic heterogeneity of *T. gondii* oocysts in cat faeces from Malaysia is outside of the scope of this study, it is an important consideration for the genetic diversity of *T. gondii* and should be considered in future research. Therefore, the PCs and FRCs surveyed in this study may play a role in the epidemiology of *T. gondii*, which requires accurate and reliable detection techniques to prevent the spread of infection. Future research should also be conducted in a different location to determine the occurrence and genetic diversity of *T. gondii* oocysts in the environment.

## Figures and Tables

**Table 1:** Comparison between copro-microscopy and copro-PCR for detecting *T. gondii* oocysts in PC and FRC faeces.

Cats	No. of Samples Examined	Copro-Microscopy Positive (%)	Copro-PCR Positive (%)	
			B1 Gene	REP Gene
PC	100	2 (2.0)	4 (4.0)	4 (4.0)
FRC	100	5 (5.0)	13 (13.0)	13 (13.0)
Total	200	7 (3.5)	17 (8.5)	17 (8.5)

PC; pet cat, FRC, free-roaming cat, PCR; polymerase chain reaction.

**Table 2:** Analysis of PC and FRC faecal samples identified by copro-microscopy and two multicopy-target copro-PCR assays, DNA sequencing, and the BLAST results.

Sample Number	Cat	Copro-Microscopy	Copro-PCR		Sequencing and BLAST Results of REP Gene (A146527)			Isolate Designation
			B1	REP	Identified Specie	% Identity	GenBank Accession Number	
2B	PC	+	+	+	*T. gondii*	95.8	KU599165	TgCtMy01
3A	FRC	+	+	+	*T. gondii*	94.4	KX963354	TgCtMy02
5A	FRC	−	+	+	*T. gondii*	93.6	MH560583	TgCtMy03
6A	FRC	+	+	+	*T. gondii*	93.6	KY628128	TgCtMy04
7A	FRC	−	+	+	*T. gondii*	100	KU873097	TgCtMy05
8B	PC	+	+	+	*T. gondii*	100	KU873097	TgCtMy06
9B	PC	−	+	+	*T. gondii*	96.7	KX963354	TgCtMy07
16B	FRC	−	+	+	*T. gondii*	95.8	DQ779188	TgCtMy08
26B	FRC	+	+	+	*T. gondii*	98.9	KX963354	TgCtMy09
27A	FRC	−	+	+	*T. gondii*	97.4	KU599165	TgCtMy10
36A	FRC	−	+	+	*T. gondii*	97.8	KX963354	TgCtMy11
36B	FRC	+	+	+	*T. gondii*	100	KU873097	TgCtMy12
37A	FRC	−	+	+	*T. gondii*	100	KX963354	TgCtMy13
40B	PC	−	+	+	*T. gondii*	97.8	KU599811	TgCtMy14
42B	FRC	−	+	+	*T. gondii*	97.8	KX963354	TgCtMy15
50A	FRC	+	+	+	*T. gondii*	89.7	DQ779188	TgCtMy16

C, pet cat; FRC, free-roaming cat; +, positive; −, negative; Tg, *T. gondii*; Ct, cat; My, Malaysia, F, forward; R, reverse primers.

**Table 3:** Primer sequence used for the amplification of B1 and REP genes from oocysts of *T. gondii* in cat faeces.

Target Gene	Primer Sequence	Amplicon Size	PCR Cycles	Annealing Temperature	GenBank Accession no.	Reference
B1 gene	**F** (5′ GGA ACT GCA TCC GTT CAT GAG 3′) **R** (5′ TCT TTA AAG CGT TCG TGG TC 3′)	194 bp	35	57 °C	AF179871	[67]
REP gene	**F** (5′ AGG CGA GGG TGA GGA TGA 3′) **R** (5′ TCG TCT CGT CTG GAT CGC AT 3′)	134 bp	35	62.8 °C	AF146527	[68]

## References

[1] Dubey J., Jones J. (2008). Toxoplasma gondii infection in humans and animals in the United States. Int. J. Parasitol..

[2] Tenter A.M., Heckeroth A.R., Weiss L.M. (2000). Toxoplasma gondii: From animals to humans. Int. J. Parasitol..

[3] Tyebji S., Seizova S., Hannan A.J., Tonkin C.J. (2019). Toxoplasmosis: A pathway to neuropsychiatric disorders. Neurosci. Biobehav. Rev..

[4] Mohamed Z., Hajissa K. (2016). Seroprevalence of Toxoplasma gondii infection among patients in Hospital Universiti Sains Malaysia. Trop. Biomed..

[5] Sahimin N., Lim Y.A.L., Ariffin F., Behnke J.M., Basáñez M.-G., Walker M., Lewis J.W., Noordin R., Abdullah K.A., Mohd Zain S.N. (2017). Socio-demographic determinants of Toxoplasma gondii seroprevalence in migrant workers of Peninsular Malaysia. Parasit Vectors.

[6] Chemoh W., Farhana N., Noor A. (2019). Prevalence and risk factors of Toxoplasma infection—An update in Malaysian pregnant women. Trop. Biomed..

[7] Hill D.E., Chirukandoth S., Dubey J.P. (2005). Biology and epidemiology of Toxoplasma gondii in man and animals. Anim. Health Res. Rev..

[8] Dumètre A., Dardé M.-L. (2003). How to detect Toxoplasma gondii oocysts in environmental samples?. FEMS Microbiol. Rev..

[9] Dubey J.P., Dubey J.P. (2010). Toxoplasmosis of Animals and Humans.

[10] Robert-Gangneux F., Darde M.-L. (2012). Epidemiology of and Diagnostic Strategies for Toxoplasmosis. Clin. Microbiol. Rev..

[11] Frenkel J., Dubey J., Miller N.L. (1970). Toxoplasma gondii in cats: Fecal stages identified as coccidian oocysts. Science.

[12] Chaichan P., Mercier A., Galal L., Mahittikorn A., Ariey F., Morand S., Boumédiène F., Udonsom R., Hamidovic A., Murat J.B. (2017). Geographical distribution of Toxoplasma gondii genotypes in Asia: A link with neighboring continents. Infect. Genet. Evol..

[13] Chan B.T.E., Amal R.N., Noor Hayati M.I., Kino H., Anisah N., Norhayati M., Sulaiman O., Mohammed Abdullah M., Fatmah M.S., Roslida A.R. (2008). Seroprevalence of toxoplasmosis among migrant workers from different Asian countries working in Malaysia. Southeast Asian J. Trop. Med. Public Health.

[14] van Enter B.J.D., Lau Y.-L., Ling C.L., Watthanaworawit W., Sukthana Y., Lee W.-C., Nosten F., McGready R. (2017). Seroprevalence of Toxoplasma gondii Infection in Refugee and Migrant Pregnant Women along the Thailand-Myanmar Border. Am. J. Trop. Med. Hyg..

[15] Afonso E., Germain E., Poulle M.-L., Ruette S., Devillard S., Say L., Villena I., Aubert D., Gilot-Fromont E. (2013). Environmental determinants of spatial and temporal variations in the transmission of Toxoplasma gondii in its definitive hosts. Int. J. Parasitol. Parasites Wildl..

[16] Berger-Schoch A.E., Herrmann D.C., Schares G., Müller N., Bernet D., Gottstein B., Frey C.F. (2011). Prevalence and genotypes of Toxoplasma gondii in feline faeces (oocysts) and meat from sheep, cattle and pigs in Switzerland. Vet. Parasitol..

[17] Chemoh W., Sawangjaroen N., Nissapatorn V., Sermwittayawong N. (2016). Molecular investigation on the occurrence of Toxoplasma gondii oocysts in cat feces using TOX-element and ITS-1 region targets. Vet. J..

[18] Dabritz A.A., Miller M.A., Atwill E.R., Gardner I.A., Leutenegger C.M., Melli A.C., Conrad P.A. (2007). Detection of Toxoplasma gondii-like oocysts in cat feces and estimates of the environmental oocyst burden. J. Am. Vet. Med Assoc..

[19] Herrmann D.C., Pantchev N., Vrhovec M.G., Barutzki D., Wilking H., Fröhlich A., Lüder C.G.K., Conraths F.J., Schares G. (2010). Atypical Toxoplasma gondii genotypes identified in oocysts shed by cats in Germany. Int. J. Parasitol..

[20] Mancianti F., Nardoni S., Ariti G., Parlanti D., Giuliani G., Papini R.A. (2010). Cross-sectional survey of Toxoplasma gondii infection in colony cats from urban Florence (Italy). J. Feline Med. Surg..

[21] Schares G., Vrhovec M.G., Pantchev N., Herrmann D.C., Conraths F.J. (2008). Occurrence of Toxoplasma gondii and Hammondia hammondi oocysts in the faeces of cats from Germany and other European countries. Vet. Parasitol..

[22] Jokelainen P., Simola O., Rantanen E., Näreaho A., Lohi H., Sukura A. (2012). Feline toxoplasmosis in Finland: Cross-sectional epidemiological study and case series study. J. Vet. Diagn. Investig..

[23] dos Santos T.R., Nunes C.M., Luvizotto M.C.R., de Moura A.B., Lopes W.D.Z., da Costa A.J., Bresciani K.D.S. (2010). Detection of Toxoplasma gondii oocysts in environmental samples from public schools. Vet. Parasitol..

[24] Aramini J.J., Stephen C., Dubey J.P., Engelstoft C., Schwantje H., Ribble C.S. (1999). Potential contamination of drinking water with Toxoplasma gondii oocysts. Epidemiol. Infect..

[25] Dubey J.P., Zhu X.Q., Sundar N., Zhang H., Kwok O.C.H., Su C. (2007). Genetic and biologic characterization of Toxoplasma gondii isolates of cats from China. Vet. Parasitol..

[26] Salant H., Hamburger J., Spira D.T. (2010). A Comparative Analysis of Coprologic Diagnostic Methods for Detection of Toxoplama gondii in Cats. Am. J. Trop. Med. Hyg..

[27] Lilly E.L., Wortham C.D. (2013). High prevalence of Toxoplasma gondii oocyst shedding in stray and pet cats (Felis catus) in Virginia, United States. Parasites Vectors.

[28] Veronesi F., Santoro A., Milardi G.L., Diaferia M., Morganti G., Ranucci D., Gabrielli S. (2017). Detection of Toxoplasma gondii in faeces of privately owned cats using two PCR assays targeting the B1 gene and the 529-bp repetitive element. Parasitol. Res..

[29] Saitou N., Nei M. (1987). The neighbor-joining method: A new method for reconstructing phylogenetic trees. Mol. Biol. Evol..

[30] Tamura K., Masatoshi N., Sudhir K. (2004). Prospects for inferring very large phylogenies by using the neighbor-joining method. Proc. Natl. Acad. Sci. USA.

[31] Kumar S., Stecher G., Li M., Knyaz C., Tamura K. (2018). MEGA X: Molecular Evolutionary Genetics Analysis across Computing Platforms. Mol. Biol. Evol..

[32] Abd El-Razik K.A., Barakat A.M.A., Hussein H.A., Younes A.M., Elfadaly H.A., Eldebaky H.A., Soliman Y.A. (2018). Seroprevalence, isolation, molecular detection and genetic diversity of Toxoplasma gondii from small ruminants in Egypt. J. Parasit. Dis..

[33] Ahmad A.F., Ngui R., Muhammad Aidil R., Lim Y.A.L., Rohela M. (2014). Current status of parasitic infections among Pangkor Island community in Peninsular Malaysia. Trop. Biomed..

[34] Andiappan H., Nissapatorn V., Sawangjaroen N., Nyunt M.H., Lau Y.-L., Khaing S.L., Aye K.M., Mon N.C.N., Tan T.-C., Kumar T. (2014). Comparative study on Toxoplasma infection between Malaysian and Myanmar pregnant women. Parasites Vectors.

[35] Andiappan H., Nissapatorn V., Sawangjaroen N., Khaing S.-L., Salibay C.C., Cheung M.M.M., Dungca J.Z., Chemoh W., Xiao Teng C., Lau Y.-L. (2014). Knowledge and practice on Toxoplasma infection in pregnant women from Malaysia, Philippines, and Thailand. Front. Microbiol..

[36] Brandon-Mong G.-J., Che Mat Seri N.A.A., Sharma R.S.-K., Andiappan H., Tan T.-C., Lim Y.A.-L., Nissapatorn V. (2015). Seroepidemiology of Toxoplasmosis among People Having Close Contact with Animals. Front. Immunol..

[37] Cantlay J.C., Ingram D.J., Meredith A.L. (2017). A Review of Zoonotic Infection Risks Associated with the Wild Meat Trade in Malaysia. EcoHealth.

[38] Chandrawathani P., Nurulaini R., Zanin C.M., Premaalatha B., Adnan M., Jamnah O., Khor S.K., Khadijah S., Lai S.Z., Shaik M.A.B. (2008). Seroprevalence of Toxoplasma gondii antibodies in pigs, goats, cattle, dogs and cats in peninsular Malaysia. Trop. Biomed..

[39] Emelia O., Rahana A.R., Mohamad Firdaus A., Cheng H.S., Nursyairah M.S., Fatinah A.S., Azmawati M.N., Siti N.A.M., Aisah M.Y. (2014). IgG avidity assay: A tool for excluding acute toxoplasmosis in prolonged IgM titer sera from pregnant women. Trop. Biomed..

[40] Fazly Z., Nurulaini R., Shafarin M., Fariza N., Zawida Z., Muhamad H., Adnan M., Premaalatha B., Erwanas A., Zaini C. (2013). Zoonotic parasites from exotic meat in Malaysia. Trop. Biomed..

[41] Normaznah Y., Azizah M.A., Azuan M.I., Latifah I., Rahmat S., Nasir M.A. (2015). Seroprevalence of Toxoplasma gondii in Rodents from Various Locations in Peninsular Malaysia. Southeast Asian J. Trop. Med. Public Health.

[42] Omar A., Bakar O.C., Adam N.F., Osman H., Osman A., Suleiman A.H., Manaf M.R.A., Selamat M.I. (2015). Seropositivity and serointensity of Toxoplasma gondii antibodies and DNA among patients with schizophrenia. Korean J. Parasitol..

[43] Rahman W.A., Manimegalai V., Chandrawathani P., Nurulaini R., Zaini C.M., Premaalatha B. (2011). Seroprevalence of Toxoplasma gondii in Malaysian Cattle. Malays. J. Vet. Res..

[44] Ching X.T., Fong M.Y., Lau Y.L. (2016). Evaluation of Immunoprotection Conferred by the Subunit Vaccines of GRA2 and GRA5 against Acute Toxoplasmosis in BALB/c Mice. Front. Microbiol..

[45] Emelia O., Zeehaida M., Sulaiman O., Rohela M., Saadatnia G., Yeng C., Rahmah N. (2009). An Assay for Selection of Sera with Circulating Toxoplasma gondii Antigens. J. Immunoass. Immunochem..

[46] Hajissa K., Zakaria R., Suppian R., Mohamed Z. (2017). An evaluation of a recombinant multiepitope based antigen for detection of Toxoplasma gondii specific antibodies. BMC Infect. Dis..

[47] Ling Lau Y., Yik Fong M. (2008). Toxoplasma gondii: Serological characterization and immunogenicity of recombinant surface antigen 2 (SAG2) expressed in the yeast Pichia pastoris. Exp. Parasitol..

[48] Saadatnia G., Ghaffarifar F., Khalilpour A., Amerizadeh A., Rahmah N. (2011). Saadatnia et al_ 2011_A Toxoplasma gondii 10 kDa in vitro excretory secretory antigen reactive with human IgM and IgA antibodies.pdf. Trop. Biomed..

[49] Puvanesuaran V.R., Noordin R., Balakrishnan V. (2013). Genotyping of Toxoplasma gondii Isolates from Wild Boars in Peninsular Malaysia. PLoS ONE.

[50] Puvanesuaran V.R., Noordin R., Balakrishnan V. (2013). Isolation and Genotyping of Toxoplasma gondii from Free-Range Ducks in Malaysia. Avian Dis..

[51] Kolören Z., Dubey J.P. (2019). A review of toxoplasmosis in humans and animals in Turkey. Parasitology.

[52] Shapiro K., Bahia-Oliveira L., Dixon B., Dumètre A., de Wit L.A., VanWormer E., Villena I. (2019). Environmental transmission of Toxoplasma gondii: Oocysts in water, soil and food. Food Waterborne Parasitol..

[53] Afonso E., Lemoine M., Poulle M.-L., Ravat M.-C., Romand S., Thulliez P., Villena I., Aubert D., Rabilloud M., Riche B. (2008). Spatial distribution of soil contamination by Toxoplasma gondii in relation to cat defecation behaviour in an urban area. Int. J. Parasitol..

[54] Gotteland C., Gilot-Fromont E., Aubert D., Poulle M.-L., Dupuis E., Dardé M.-L., Forin-Wiart M.-A., Rabilloud M., Riche B., Villena I. (2014). Spatial distribution of Toxoplasma gondii oocysts in soil in a rural area: Influence of cats and land use. Vet. Parasitol..

[55] Lass A., Pietkiewicz H., Modzelewska E., Dumètre A., Szostakowska B., Myjak P. (2009). Detection of Toxoplasma gondii oocysts in environmental soil samples using molecular methods. Eur. J. Clin. Microbiol. Infect. Dis..

[56] Al-Kappany Y.M., Rajendran C., Ferreira L.R., Kwok O.C.H., Abu-Elwafa S.A., Hilali M., Dubey J.P. (2010). High Prevalence of Toxoplasmosis in Cats from Egypt: Isolation of Viable Toxoplasma gondii, Tissue Distribution, and Isolate Designation. J. Parasitol..

[57] Stojanovic V., Foley P. (2011). Infectious disease prevalence in a feral cat population on Prince Edward Island, Canada. Can. Vet. J..

[58] Dubey J.P., Choudhary S., Tilahun G., Tiao N., Gebreyes W.A., Zou X., Su C. (2013). Genetic diversity of Toxoplasma gondii isolates from Ethiopian feral cats. Vet. Parasitol..

[59] Dubey J.P., Su C., Cortés J.A., Sundar N., Gomez-Marin J.E., Polo L.J., Zambrano L., Mora L.E., Lora F., Jimenez J. (2006). Prevalence of Toxoplasma gondii in cats from Colombia, South America and genetic characterization of T. gondii isolates. Vet. Parasitol..

[60] Karatepe B., Babür C., Karatepe M., Kiliç S., Dündar B. (2008). Prevalence of Toxoplasma gondii antibodies and intestinal parasites in stray cats from Nigde, Turkey. Ital. J. Anim. Sci..

[61] Miró G., Montoya A., Jiménez S., Frisuelos C., Mateo M., Fuentes I. (2004). Prevalence of antibodies to Toxoplasma gondii and intestinal parasites in stray, farm and household cats in Spain. Vet. Parasitol..

[62] Qian W., Wang H., Su C., Shan D., Cui X., Yang N., Lv C., Liu Q. (2012). Isolation and characterization of Toxoplasma gondii strains from stray cats revealed a single genotype in Beijing, China. Vet. Parasitol..

[63] Tavalla M., Asgarian F., Kazemi F. (2017). Prevalence and genetic diversity of Toxoplasma gondii oocysts in cats of southwest of Iran. Infect. Dis. Health.

[64] Du F., Feng H.L., Nie H., Tu P., Zhang Q.L., Hu M., Zhou Y.Q., Zhao J.L. (2012). Survey on the contamination of Toxoplasma gondii oocysts in the soil of public parks of Wuhan, China. Vet. Parasitol..

[65] Dubey J.P. (2006). Comparative infectivity of oocysts and bradyzoites of Toxoplasma gondii for intermediate (mice) and definitive (cats) hosts. Vet. Parasitol..

[66] Lass A., Pietkiewicz H., Szostakowska B., Myjak P. (2012). The first detection of Toxoplasma gondii DNA in environmental fruits and vegetables samples. Eur. J. Clin. Microbiol. Infect. Dis..

[67] Burg J.L. (1989). Direct and Sensitive Detection of a Pathogenic Protozoan, Toxoplasma gondii, by Polymerase Chain Reaction. J. Clin. Microbiol..

[68] Homan W.L., Vercammen M., Braekeleer J.D., Verschueren H. (2000). Identification of a 200- to 300-fold repetitive 529 bp DNA fragment in Toxoplasma gondii, and its use for diagnostic and quantitative PCRp. Int. J. Parasitol..

[69] Jung B.-K., Lee S.-E., Lim H., Cho J., Kim D.-G., Song H., Kim M.-J., Shin E.-H., Chai J.-Y. (2015). Toxoplasma gondii B1 Gene Detection in Feces of Stray Cats around Seoul, Korea and Genotype Analysis of Two Laboratory-Passaged Isolates. Korean J. Parasitol..

[70] Jones J.L., Dubey J.P. (2010). Waterborne toxoplasmosis—Recent developments. Exp. Parasitol..

[71] Salant H., Markovics A., Spira D.T., Hamburger J. (2007). The development of a molecular approach for coprodiagnosis of Toxoplasma gondii. Vet. Parasitol..

[72] Dabritz H.A., Conrad P.A. (2010). Cats and Toxoplasma: Implications for Public Health. Zoonoses Public Health.

[73] Simon J.A., Kurdzielewicz S., Jeanniot E., Dupuis E., Marnef F., Aubert D., Villena I., Poulle M.-L. (2017). Spatial distribution of soil contaminated with Toxoplasma gondii oocysts in relation to the distribution and use of domestic cat defecation sites on dairy farms. Int. J. Parasitol..

[74] Torrey E.F., Yolken R.H. (2013). Toxoplasma oocysts as a public health problem. Trends Parasitol..

[75] VanWormer E., Fritz H., Shapiro K., Mazet J.A., Conrad P.A. (2013). Molecules to modeling: Toxoplasma gondii oocysts at the human–animal–environment interface. Comp. Immunol. Microbiol. Infect. Dis..

[76] Khan A., Dubey J.P., Su C., Ajioka J.W., Rosenthal B.M., Sibley L.D. (2011). Genetic analyses of atypical Toxoplasma gondii strains reveal a fourth clonal lineage in North America. Int. J. Parasitol..

[77] Velmurugan G.V., Dubey J.P., Su C. (2008). Genotyping studies of Toxoplasma gondii isolates from Africa revealed that the archetypal clonal lineages predominate as in North America and Europe. Vet. Parasitol..

[78] Bahadori E.S., Sadraei J., Dalimi A., Namroodi S., Pirestani M. (2018). Phylogenetic Analysis of Toxoplasma gondii Type II and Type III by PCRRFLP Plus Sequencing on Wild-Rats of Golestan Forest, Iran. J. Vet. Sci. Technol..

[79] Li M., Mo X.-W., Wang L., Chen H., Luo Q.-L., Wen H.-Q., Wei W., Zhang A.-M., Du J., Lu F.-L. (2014). Phylogeny and virulence divergency analyses of Toxoplasma gondii isolates from China. Parasites Vectors.

[80] Pena H.F.J., Gennari S.M., Dubey J.P., Su C. (2008). Population structure and mouse-virulence of Toxoplasma gondii in Brazil. Int. J. Parasitol..

[81] Landis J.R., Koch G.G. (1977). The Measurement of Observer Agreement for Categorical Data. Biometrics.

